# Supplementary material for: Factors associated with in-hospital mortality of patients admitted to an intensive care unit in a tertiary hospital in Malawi
Source: PLoS One. 2022 Sep 30;17(9):e0273647. doi: 10.1371/journal.pone.0273647 (PMC9524689; doi:10.1371/journal.pone.0273647)
Supplement: S1 Table — (DOCX) [file pone.0273647.s001.docx]

**Supplementary table 1. Sensitivity analyses of the factors associated with in-hospital mortality when missing data are handled by i) only including patients with complete data and ii) imputed as deranged**

| Factor | ONLY INCLUDING PATIENTS WITH COMPLETE DATA | | | | | | | | | | MISSING DATA IMPUTTED AS DERANGED | | | | | | | | | | |
| --- | --- | --- | --- | --- | --- | --- | --- | --- | --- | --- | --- | --- | --- | --- | --- | --- | --- | --- | --- | --- | --- |
|  | odds ratio | P - value | 95% CI | Sensitivity (%) | Specificity (%) | PPV (%) | NPV (%) | Adj Odds Ratio | P - Value | 95% CI | odds ratio |  | P - value | 95% CI | Sensitivity (%) | Specificity | PPV | NPV (%) | Adj Odds Ratio | P - Value | 95% CI |
| Any severly deranged vital sign  N = 398 | 3.4 | <0.001 | 2.0-5.8 | 93 | 21 | 52 | 76 | 3.1 | <0.001 | 1.8-5.3 | 2.5 |  | <0.001 | 1.8-4.7 | 94 | 16 | 48 | 76 | 2.8 | <0.001 | 1.7-4.7 |
| Treatment with inotrope or vasopressor  (N=779) | 3.6 | <0.001 | 2.4-2.5 | 48 | 80 | 73 | 57 | 3.0 | 0.158 | 0.7-13.3 | 2.9 |  | <0.001 | 3.3-6.6 | 63 | 73 | 78 | 57 | 2.8 | 0.000 | 1.8-4.2 |
| Received cardiopulmonary resuscitation  (N=794) | 2.4 | <0.001 | 1.6-3.6 | 41 | 78 | 66 | 56 | 1.9 | 0.434 | 0.4-10.1 | 2.3 |  | <0.001 | 2.9-6.0 | 60 | 74 | 77 | 56 | 2.1 | 0.001 | 1.4-3.3 |
| Treatment with mechanical ventilation  (n=794) | 1.6 | 0.012 | 1.1- 2.4 | 80 | 29 | 62 | 50 | 4.2 | 0.068 | 0.9-19.8 | 1.9 |  | 0.012 | 1.1-2.4 | 81 | 28 | 62 | 50 | 1.2 | 0.546 | 0.7-1.9 |
| HIV status  (N-114) | 0.8 | 0.656 | 0.4-1.9 | 26 | 70 | 48 | 47 | 0.3 | 0.137 | 0.04-1.5 | 1.1 |  | 0.200 | 0.8-2.2 | 88 | 15 | 61 | 50 | 0.6 | 0.127 | 0.4-1.1 |
| Capillary refill time > 3 seconds  N=409) | 1.2 | 0.552 | 0.6-2.3 | 12 | 90 | 52 | 53 | 0.9 | 0.892 | 0.2-4.5 | 1.1 |  | <0.001 | 2.3-5.0 | 55 | 73 | 75 | 53 | 1.9 | 0.002 | 1.2-2.9 |
| Sex (Male)  (N=822) | 0.8 | 0.225 | 0.6 - 1.1 | 47 | 46 | 56 | 37 | 0.9 | 0.421 | 0.7-1.2 |  |  |  |  |  |  |  |  |  |  |  |
| Having surgery  (N=803) | 0.8 | 0.120 | 0.5-1.1 | 58 | 36 | 57 | 37 | 0.6 | 0.422 | 0.2-1.5 | 0.6 |  | 0.158 | 0.6-1.1 | 59 | 35 | 57 | 40 | 0.6 | 0.010 | 0.4-0.9 |
| Age group >50  (N=822) | 1.5 | 0.114 | 1.1-2.2 | 24 | 82 | 66 | 42 | 1.4 | 0.059 | 1.0-2.1 |  |  |  |  |  |  |  |  | 1.3 | 0.308 | 0.8-2 |
| Emergency Admission  N=788) | 2.2 | 0.002 | 1.3-3.7 | 92 | 17 | 62 | 58 | 1.4 | 0.735 | 0.2-8.5 | 1.1 |  | 0.002 | 1.3-3.7 | 92 | 16 | 62 | 58 | 1.5 | 0.177 | 0.8-2.6 |
| Severly deranged temperature  (N-738) | 1.1 | 0.424 | 0.8-1.6 | 47 | 57 | 59 | 44 | 0.3 | 0.055 | 0.6 - 1.0 | 1.2 |  | 0.065 | 1.0-1.9 | 53 | 55 | 63 | 44 | 1.2 | 0.377 | 0.8-1.7 |
