# Supplementary material for: Factors associated with in-hospital mortality of patients admitted to an intensive care unit in a tertiary hospital in Malawi
Source: PLoS One. 2022 Sep 30;17(9):e0273647. doi: 10.1371/journal.pone.0273647 (PMC9524689; doi:10.1371/journal.pone.0273647)
Supplement: S2 Table — (DOCX) [file pone.0273647.s002.docx]

**Supplementary Table 2. Description of models used in the study**

| **Model** | **Population and location of people used to derive model** | **Component variables** |
| --- | --- | --- |
| Malawi intensive care mortality risk  evaluation model (MIME) | ICU patients, Malawi | Age, sex, admitting service, systolic pressure, mental status, and  fever (38.4C) during the ICU course |
| Quick Sequential Organ Failure  Assessment (qSOFA) | ICU patients | Respiratory rate, systolic blood pressure, and mental status |
| Universal Vital Assessment (UVA) | Medical wards | Temperature, heart rate, respiratory rate, systolic blood pressure,  oxygen saturation, Glasgow coma scale, HIV status |
| NEWS | UK, acute hospital settings | ([respiratory rate](about:blank), [oxygen saturation](about:blank), [temperature](about:blank), [blood pressure](about:blank), [pulse](about:blank)/[heart rate](about:blank), [AVPU response](about:blank)) |
| TROPICS | Asia, ICU patients | Emergency surgery, respiratory rate, systolic blood pressure, Glasgow coma score, blood urea, hemoglobin |
| TOTAL | Malawi, emergency department | Tachypnoea, Oxygen saturation, Temperature, Alert and Loss of independence |
