# Supplementary material for: Factors associated with in-hospital mortality of patients admitted to an intensive care unit in a tertiary hospital in Malawi
Source: PLoS One. 2022 Sep 30;17(9):e0273647. doi: 10.1371/journal.pone.0273647 (PMC9524689; doi:10.1371/journal.pone.0273647)
Supplement: S3 Table — (DOCX) [file pone.0273647.s003.docx]

**Supplementary Table 3 Univariable** **logistic regression of individual vital signs**

| Variable* | N = 822(%) | Mortality n^1^/n^2^ (%) with derangement | Mortality n^1^/n^2^ (%) without derangement | OR | 95% C.I | p-value |
| --- | --- | --- | --- | --- | --- | --- |
| Severely deranged heart rate  Severely Deranged respiratory rate**  Low conscious level  Hypoxia (oxygen saturation < 90%)  Hypotension (Systolic BP)) | 145 (17.6)  146 (17.8)  245 (29.8)  83 (10.1)  140 (17.0) | 73/145 (50.3)  68/146 (46.6)  134/245 (54.7)  58/83 (69.9)  84/140 (60) | 295/677 (43.6)  300/676 (44.4)  234/577 (40.6)  310/739(41.9)  284/682 (41.6) | 1.3  1.1  1.8  3.2  2.1 | 0.9 – 1.9  0.8 – 1.6  1.3 – 2.4  2.0 – 5.2  1.5 – 2.8 | 0.137  0.628  <0.001  <0.001  <0.001 |

* The age specific cut-offs for the individual vital signs can be seen in Figures 1 .
