# Supplementary material for: Factors associated with in-hospital mortality of patients admitted to an intensive care unit in a tertiary hospital in Malawi
Source: PLoS One. 2022 Sep 30;17(9):e0273647. doi: 10.1371/journal.pone.0273647 (PMC9524689; doi:10.1371/journal.pone.0273647)
Supplement: S4 Table — (DOCX) [file pone.0273647.s004.docx]

**S4 Table. Percentage of data missing per variable**

| Total patients 824 | Available data/completed | Percentage of data missing |
| --- | --- | --- |
| Glascow coma score (GCS) | 473 | 42% |
| Heart rate | 788 | 4% |
| Systolic blood pressure | 760 | 8% |
| Respiratory rate | 760 | 8% |
| Oxygen saturations | 784 | 5% |
| Sex | 824 | 0% |
| Age | 824 | 0% |
| Admission type | 788 | 4 % |
| Had surgery | 803 | 3% |
| Received vasopressor | 779 | 5% |
| Received CPR | 794 | 4% |
| Mechanical Ventilation | 794 | 4% |
| Specialty | 822 | 0.2% |
| Temperature | 738 | 10% |
| Capillary refill time | 409 | 50% |
| HIV status | 114 | 86% |
