# Supplementary material for: Factors associated with in-hospital mortality of patients admitted to an intensive care unit in a tertiary hospital in Malawi
Source: PLoS One. 2022 Sep 30;17(9):e0273647. doi: 10.1371/journal.pone.0273647 (PMC9524689; doi:10.1371/journal.pone.0273647)
Supplement: S5 Table — (DOCX) [file pone.0273647.s005.docx]

**Supplementary table 5: Predictive values of severity score models for patients over 16 years included in the prospective data collection period**

|  | **Number with critical score (%)**  **N = 435** | **Mortality**  **n^1^/n^2^ (%)**  **with critical score** | **Mortality**  **n^1^/n^2^ (%) without critical score** | **Odds Ratio** | **p-value** | **95% C.I** | **Sensitivity %**  **(95%C.I)** | **Specificity %**  **(95% C.I)** | **PPV %**  **(95% C.I)** | **NPV %**  **(95% C.I)** |
| --- | --- | --- | --- | --- | --- | --- | --- | --- | --- | --- |
| Any severely deranged vital sign | 308  (71) | 165/308  (54) | 50/127  (39) | 1.8 | 0.007 | 1.2-2.7 | 77  (71-82) | 35  (29-42) | 54  (48-59) | 61  (52-69) |
| NEWS Score =>7 | 347  (80) | 183/347  (53) | 32/88  (36) | 2.0 | 0.007 | 1.2-3.2 | 85  (80-90) | 26  (20-32) | 53  (47-58) | 64  (53-74) |
| qSofa  =>2 | 178  (41) | 98/178  (55) | 117/257  (46) | 1.5 | 0.051 | 0.9-2.2 | 46  (39-53) | 64  (57-70) | 55  (47-63) | 55  (48-61) |
| UVA Score  >=5 | 215  (49) | 111/215  (52) | 104/220  (47) | 1.2 | 0.364 | 0.8-1.7 | 52  (45-59) | 53  (46-70) | 52  (45-59) | 53  (46-67) |
| TOTAL Score  >=2 | 338  (78) | 173/338  (51) | 42/97  (43) | 1.4 | 0.172 | 0.9-2.2 | 81  (75-86) | 25  (19-31) | 51  (46-57) | 57  (46-67) |
| TROPICS  Score >= 8 | 40  (9) | 21/40  (53) | 194/395  (49) | 1.1 | 0.683 | 0.6-2.2 | 10  (6-15) | 91  (87-95) | 53  (36-69) | 51  (46-56) |
| MIME score  >=2 | 308  (71) | 155/308  (50) | 60/127  (47) | 1.1 | 0.559 | 0.7-1.7 | 72  (66-78) | 31  (24-37) | 50  (45-56) | 53  (44-62) |
