# Supplementary material for: Factors associated with in-hospital mortality of patients admitted to an intensive care unit in a tertiary hospital in Malawi
Source: PLoS One. 2022 Sep 30;17(9):e0273647. doi: 10.1371/journal.pone.0273647 (PMC9524689; doi:10.1371/journal.pone.0273647)
Supplement: S6 Table — (DOCX) [file pone.0273647.s006.docx]

**Supplementary table 6: Predictive values of severity score models for patients over 16 years included in the retrospective data collection period**

|  | **Number with critical score (%)**  **N = 218** | **Mortality**  **n^1^/n^2^ (%)**  **with critical score** | **Mortality**  **n^1^/n^2^ (%) without critical score** | **Odds Ratio** | **p-value** | **95% C.I** | **Sensitivity %**  **(95%C.I)** | **Specificity %**  **(95% C.I)** | **PPV %**  **(95% C.I)** | **NPV %**  **(95% C.I)** |
| --- | --- | --- | --- | --- | --- | --- | --- | --- | --- | --- |
| Any severely deranged vital sign | 110  (50) | 57/110  (52) | 28/108  (26) | 3.0 | <0.001 | 1.7-5.4 | 67  (56-77) | 60  (51-69) | 52  (42-61) | 74  (64-82) |
| NEWS Score =>7 | 138  (63) | 64/138  (46) | 21/80  (26) | 2.4 | 0.004 | 1.3-4.4 | 75  (65-84) | 44  (36-53) | 46  (38-55) | 74  (62-83) |
| qSofa  =>2 | 56  (26) | 27/56  (48) | 58/162  (36) | 1.7 | 0.102 | 0.9-3.1 | 32  (22-43) | 78  (70-85) | 48  (35-62) | 64  (56-72) |
| UVA Score  >=5 | 36  (17) | 18/36  (50) | 67/182  (37) | 1.7 | 0.141 | 0.8-3.5 | 21  (13-31) | 87  (80-92) | 50  (33-67) | 63  (56-70) |
| TOTAL Score  >=2 | 116  (53) | 51/116  (44) | 34/102  (33) | 1.6 | 0.109 | 0.9-2.7 | 61  (49-71) | 51  (42-60) | 44  (35-54) | 67  (57-76) |
| TROPICS  Score >= 8 | 2  (0.9) | 1/2  (50) | 84/216  (25) | 1.6 | 0.750 | 0.09-25.4 | 1  (0 – 6) | 99  (96-100) | 50  (1-98) | 61  (54-68) |
| MIME score  >=2 | 112  (51) | 52/112  (46) | 33/106  (31) | 1.9 | 0.021 | 1.1-3.3 | 61  (50-72) | 55  (46-64) | 46  (37-56) | 69  (59-78) |
