# Supplementary material for: Factors associated with in-hospital mortality of patients admitted to an intensive care unit in a tertiary hospital in Malawi
Source: PLoS One. 2022 Sep 30;17(9):e0273647. doi: 10.1371/journal.pone.0273647 (PMC9524689; doi:10.1371/journal.pone.0273647)
Supplement: S7 Table — (DOCX) [file pone.0273647.s007.docx]

**Supplementary Table 7: Predictive values of the scoring models for patients over 16 years with different cut offs for defining critical illness used**

| Severity model and the cut-off value used | Number with critical score (%)  N = 722 | Mortality  n^1^/n^2^ (%)  with critical score | Mortality  n^1^/n^2^ (%) without critical score | Odds Ratio | p-value | 95% C.I | AUC | Sensitivity %  (95%C.I) | Specificity %  (95% C.I) | PPV %  (95% C.I) | NPV %  (95% C.I) |
| --- | --- | --- | --- | --- | --- | --- | --- | --- | --- | --- | --- |
| NEWS Score =>2 | 721  (99) | 335/721  (46) | 0/1  (0) | 1 | 0.058 | 0.7-1.0 | 0.50 | 100  (99-100) | 0.3  (0.0-1.4) | 47  (43-50.2) | 100  (3-100) |
| NEWS Score =>3 | 713  (98) | 332/713  (47) | 3/9  (33) | 1.7 | 0.435 | 0.4-7 | 0.50 | 99  (97-100) | 2  (0.6 - 3) | 47  (43-50) | 67  (30-93) |
| NEWS Score =>4 | 695  (96) | 327/695  (47) | 8/27  (30) | 2.1 | 0.081 | 0.9-4.9 | 0.51 | 98  (95-99) | 25  (3-8) | 47  (43-51) | 70  (50-86) |
| NEWS Score =>5 | 663  (92) | 319/663  (48) | 16/59  (27) | 2.4 | 0.003 | 1.4-4.5 | 0.53 | 95  (92-97) | 11  (8-15) | 48  (44-52) | 73  (60-84) |
| NEWS Score =>6 | 609  (84) | 298/609  (50) | 37/113  (33) | 2.0 | 0.002 | 1.3-3.0 | 0.54 | 89  (85-92) | 20  (16-24) | 50  (45-53) | 67  (58-76) |
| NEWS Score =>7 | 545  (75) | 280/545  (51) | 55/177  (31) | 2.3 | <0.001 | 1.6-3.4 | 0.57 | 84  (79-87) | 32  (27-36) | 51  (47-57) | 69  (62-76) |
| NEWS Score =>8 | 454  (63) | 236/454  (52) | 99/268  (37) | 1.8 | <0.001 | 1.4-2.5 | 0.56 | 70  (65-75) | 44  (39-49) | 52  (47-57) | 63  (57-69) |
| NEWS Score =>9 | 359  (50) | 189/359  (53) | 146/363  (40) | 1.7 | 0.001 | 1.2-2.2 | 0.56 | 56  (51-62) | 56  (51-61) | 53  (47-57) | 60  (55-65) |
| NEWS Score =>10 | 287  (40) | 153/287  (53) | 182/435  (42) | 1.6 | 0.003 | 1.2-2.1 | 0.55 | 46  (40-51) | 65  (60-70) | 53  (47-59) | 58  53-63) |
| NEWS Score =>11 | 200  (28) | 109/200  (55) | 226/522  (43) | 1.6 | 0.007 | 1.1-2.1 | 0.55 | 33  (28-38) | 77  (72-81) | 55  (47-62) | 57  (53-61) |
| NEWS Score =>12 | 128  (18) | 74/128  (58) | 261/594  (44) | 1.7 | 0.005 | 1.1-2.6 | 0.54 | 22  (18-27) | 86  (82-89) | 58  (49-67) | 56  (52-60) |
| NEWS Score =>13 | 81  (11) | 47/81  (58) | 288/641  (45) | 1.7 | 0.027 | 1.1-2.7 | 0.53 | 14  (11-18) | 91  (88-94) | 58  (47-69) | 55  (51-59) |
| NEWS Score =>14 | 52  (7) | 33/52  (63) | 302/670  (45) | 2.1 | 0.012 | 1.2-3.8 | 0.53 | 10  (7-14) | 95  (92-97) | 64  (49-76) | 55  (51-59) |
| NEWS Score =>15 | 22  (3) | 15/22  (68) | 320/700  (46) | 2.5 | 0.044 | 1.0-6.3 | 0.52 | 5  (3-7) | 98  (96-99) | 68  (45-86) | 54  (51-58) |
| NEWS Score =>16 | 13  (2) | 10/13  (77) | 325/709  (46) | 3.90 | 0.039 | 1.1-14 | 0.51 | 3  (1-5) | 99  (98-100) | 77  (46-95) | 54  (50-58) |
| NEWS Score =>17 | 9  (1) | 6/9  (66) | 329/713  (46) | 2.3 | 0.233 | 0.6-9.4 | 0.50 | 2  (0.7-3.9) | 99  (98-100) | 67  (30-93) | 54  (50-58) |
| NEWS Score =>18 | 3  (0.4) | 1/3  (33) | 334/719  (46) | 0.6 | 0.653 | 0.1-6.3 | 0.50 | 0.3  (0 - 2) | 99  (98-100) | 33  (0.8-91) | 54  (50-58) |
| qSofa  =>1 | 613  (85) | 298/613  (49) | 37/109  (34) | 1.8 | 0.005 | 1.2-2.8 | 0.53 | 89  (85-92) | 19  (15-23) | 49  (45-53) | 66  (56-75) |
| qSofa  =>2 | 267  (34) | 147/267  (55) | 188/455  (42) | 1.7 | <0.001 | 1.3-2.4 | 0.56 | 44  (39-49) | 69  (64-74) | 55  (49-61) | 59  (54-63) |
| qSofa  =>3 | 41  (6) | 27/41  (66) | 308/681  (45) | 2.3 | 0.012 | 1.2-4.5 | 0.52 | 8  (5-12) | 96  (94-98) | 66  (49-80) | 55  (51-59) |
| UVA Score  >=1 | 633  (88) | 305/633  (48) | 30/89  (34) | 1.8 | 0.011 | 1.1-2.9 | 0.53 | 91  (88-94) | 15  (12-19) | 48  (44-52) | 66  (56-76) |
| UVA Score  >= 2 | 560  (78) | 278/560  (50) | 57/162  (35) | 1.8 | 0.001 | 1.3-2.6 | 0.55 | 83  (79-87) | 27  (23-32) | 50  (45-54) | 65  (57-72) |
| UVA Score  >= 3 | 473  (66) | 239/473  (51) | 96/249  (39) | 1.6 | 0.002 | 1.2-2.2 | 0.56 | 71  (66-76) | 40  (35-45) | 51  (46-55) | 61  (55-68) |
| UVA Score  >= 4 | 399  (55) | 204/399  (51) | 131/323  (41) | 1.5 | 0.005 | 1.1-2.1 | 0.56 | 61  (55-66) | 50  (45-55) | 51  (46-56) | 59  (54-65) |
| UVA Score  >=5 | 296  (41) | 154/296  (52) | 181/426  (43) | 1.5 | 0.012 | 1.1-2.0 | 0.55 | 46  (41-52) | 63  (58 -68) | 52  (46-58) | 58  (53-62) |
| UVA Score  >=6 | 222  (31) | 109/222  (49) | 226/500  (45) | 1.2 | 0.333 | 0.9-1.6 | 0.52 | 33  (28 -38) | 71  (66-75) | 49  (42 -56) | 55  (50 -59) |
| UVA Score  >=7 | 114  (16) | 61/114  (54) | 274/608  (45) | 1.4 | 0.098 | 0.9-2.1 | 0.53 | 18  (14-23) | 86  (83-90) | 54  (44-63) | 55  (51-59) |
| UVA Score  >=8 | 49  (7) | 28/49  (57) | 307/673  (46) | 1.6 | 0.121 | 0.9-2.9 | 0.51 | 8  (6-12) | 95  (92-97) | 57  (42-71) | 54  (51-58) |
| UVA Score  >=9 | 21  (3) | 15/21  (71) | 320/701  (46) | 3.0 | 0.026 | 1.1-7.8 | 0.51 | 5  (3-7) | 98  (97 -99) | 71  (48-89) | 54  (51-58) |
| UVA Score  >=10 | 8  (1) | 5/8  (63) | 330/714  (46) | 1.9 | 0.367 | 0.5-8.1 | 0.50 | 2  (1-3) | 99  (98-100) | 63  (25-92) | 54  (50-58) |
| TOTAL Score  >=2 | 517  (72) | 258/517  (50) | 77/205  (38) | 1.7 | 0.003 | 1.2-2.3 | 0.55 | 77  (72-81) | 33  (28-38) | 50  (46-54) | 62  (55-69) |
| TOTAL Score  >=3 | 269  (37) | 138/269  (51) | 197/453  (43) | 1.4 | 0.042 | 1.0-1.9 | 0.54 | 41  (36-47) | 66  (61-71) | 51  (45-57) | 57  (52-61) |
| TOTAL Score  >=4 | 140  (19) | 76/140  (54) | 259/582  (45) | 1.5 | 0.038 | 1.0-2.1 | 0.53 | 22  (18 -28) | 84  (79 -87) | 54  (46-63) | 56  (51-60) |
| TOTAL Score  >=5 | 17  (2) | 9/17  (53) | 326/705  (46) | 1.3 | 0.585 | 0.5-3.4 | 0.50 | 3  (1-5) | 98  (96-99) | 53  (28-77) | 54  (50 -58) |
| TROPICS  Score >= 1 | 637  (88) | 292/637  (46) | 43/85  (51) | 0.8 | 0.410 | 0.5-1.3 | 0.49 | 87  (83-91) | 11  (8-14) | 46  (42-50) | 49  (38-61) |
| TROPICS  Score >= 2 | 460  (64) | 219/460  (54) | 116/262  (44) | 1.1 | 0.388 | 0.8-1.6 | 0.52 | 65  (60-71) | 38  (33-43) | 48  (43-52) | 56  (50-62) |
| TROPICS  Score >= 3 | 406  (58) | 196/406  (48) | 139/316  (44) | 1.2 | 0.252 | 0.8-1.6 | 0.52 | 59  (53-64) | 46  (41-51) | 48  (43-53) | 56  (50-62) |
| TROPICS  Score >= 4 | 306  (42) | 157/306  (51) | 178/416  (43) | 1.4 | 0.024 | 1.0-1.9 | 0.53 | 47  (41-52) | 62  (56-66) | 51  (46-57) | 57  (52-62) |
| TROPICS  Score >= 5 | 229  (32) | 124/229  (54) | 211/493  (43) | 1.6 | 0.005 | 1.2-2.1 | 0.54 | 37  (32-42) | 73  (68-77) | 54  (48 – 61) | 57  (53-62) |
| TROPICS  Score >= 6 | 166  ((23) | 92/166  (55) | 243/556  (44) | 1.6 | 0.008 | 1.1-2.3 | 0.54 | 28  (23-33) | 81  (77-85) | 55  (48 -63) | 56  (52-61) |
| TROPICS  Score >= 7 | 100  (14) | 50/100  (50) | 285/622  (46) | 1.1 | 0.437 | 0.8-1.8 | 0.51 | 15  (11-19) | 87  (83-90) | 50  (40-60) | 54  (50-58) |
| TROPICS  Score >= 8 | 58  (8) | 32/58  (55) | 303/664  (46) | 1.5 | 0.164 | 0.9-2.5 | 0.51 | 10  (7-13) | 93  (90-96) | 55  (42 -68) | 54  (51-58) |
| TROPICS  Score >= 9 | 27  (3) | 20/27  (74) | 315/695  (45) | 3.4 | 0.005 | 1.4-8.2 | 0.52 | 6  (6-9) | 98  (96-99) | 74  (54-89) | 55  (51-58) |
| TROPICS  Score >= 10 | 12  (2) | 9/12  (75) | 325/709  (46) | 3.5 | 0.059 | 1.0-13.2 | 0.51 | 3  (1-5) | 99  (98-100) | 75  (43-95) | 54  (50 -58) |
| TROPICS  Score >= 11 | 6  (0.8) | 6/6  (100) | 329/716  (46) | 1 |  |  | 0.51 | 2  (1-4) | 100  (99-100) | 100  (54-100) | 54  (50-58) |
| TROPICS  Score >= 12 | 5  (0.6) | 5/5  (100) | 330/717  (46) | 1 |  |  |  | 2  (1-3) | 100  (99-100) | 100  (48-100) | 54  (50-58) |
| MIME score  >=1 | 690  (96) | 324/690  (47) | 11/32  (34) | 1.7 | 0.167 | 0.8-3.6 | 0.51 | 97  (94-98) | 5  (3-8) | 47  (43-51) | 66  (47-81) |
| MIME score  >=2 | 474  (66) | 234/474  (49) | 101/248  (41) | 1.4 | 0.027 | 1.0-1.9 | 0.54 | 70  (65-75) | 38  (33-43) | 49  (44-54) | 59  (53-65) |
| MIME score  >=3 | 203  (28) | 116/203  (57) | 219/519  (42) | 1.8 | 0.000 | 1.3-2.5 | 0.56 | 35  (30-40) | 78  (73-82) | 57  (50-64) | 58  (53-62) |
| MIME score  >=4 | 45  (6) | 29/45  (64) | 306/677  45 | 2.2 | 0.014 | 1.2-4.1 | 0.52 | 9  (6-12) | 96  (94-98) | 64  (49-78) | 55  (51-59) |
| MIME score  >=5 | 5  (0.6) | 4/5  (80) | 331/717  (46) | 4.7 | 0.169 | 0.5-42 | 0.50 | 1  (0-3) | 100 (99-100) | 80  (28-100) | 54  (50-58) |

*
