# Supplementary material for: Factors associated with in-hospital mortality of patients admitted to an intensive care unit in a tertiary hospital in Malawi
Source: PLoS One. 2022 Sep 30;17(9):e0273647. doi: 10.1371/journal.pone.0273647 (PMC9524689; doi:10.1371/journal.pone.0273647)
Supplement: S8 Table — (DOCX) [file pone.0273647.s008.docx]

**Supplementary Table 8. Predictive values of severity score models for patients over 16 years using cases with complete data only**

|  | **Sensitivity %**  **(95%C.I)** | **Specificity %**  **(95% C.I)** | **PPV %**  **(95% C.I)** | **NPV %**  **(95% C.I)** | **AUC** |
| --- | --- | --- | --- | --- | --- |
| Any severely deranged vital sign  N=398 | 89  (84 – 93) | 28  (22 – 35) | 52  (47 – 58) | 75  (64 – 84) | 0.59 |
| NEWS Score =>7  N=398 | 94  (90-97) | 21  (16 -27) | 52  (46-57) | 80  (68–09) | 0.58 |
| qSofa  =>2  N=425 | 53  (46-60) | 61  (54-67) | 55  (48-62) | 59  (53-66) | 0.57 |
| UVA Score  >=5  N = 81 | 69  (52-83)0 | 45  (30-61) | 54  (39- 68) | 61  (42-78) | 0.57 |
| TOTAL Score  >=2  N = 413 | 96  (93-98) | 15  (11-21) | 50  (45 -56) | 85  (07 -94) | 0.56 |
| TROPICS  Score >= 8  N = 27 | 63  (38-84) | 63  (25 – 92) | 80  (52 – 96) | 42  (52 – 96) | 0.63 |
| MIME score  >=2  N= 416 | 83  (76 -88) | 22  (17-28) | 48  (43-54) | 59  (48-70) | 0.52 |

NEWS- . National Early Warning Score; qSOFA - quick Sequential Organ Failure Assessment UVA - Universal Vital signs Assessment (UVA); TOTAL score (Tachypnoea, Oxygen saturation, Temperature, Alert and Loss of independence); TROPICS - Tropical Intensive Care Score; MIME - Malawi Intensive care Mortality risk Evaluation model
