# Supplementary material for: Factors associated with in-hospital mortality of patients admitted to an intensive care unit in a tertiary hospital in Malawi
Source: PLoS One. 2022 Sep 30;17(9):e0273647. doi: 10.1371/journal.pone.0273647 (PMC9524689; doi:10.1371/journal.pone.0273647)
Supplement: Attachment — Submitted filename: Response to Reviewers.docx [file pone.0273647.s009.docx]

Dr Mtisunge Kachingwe
Queen Elizabeth Central hospital
P.O Box 95, Blantyre, Malawi

22/12/2021

Dear Editorial board members,

**RE: Response to reviewers**

We thank the editor and the two reviewers for their comments on our manuscript titled “Factors associated with in-hospital mortality of patients admitted to an Intensive Care Unit in a tertiary hospital in Malawi”. Below is our response to each point raised by the academic editor and reviewers. We hope that we satisfyingly addressed them and that the manuscript will be now suited for publication.

Sincerely,

On behalf of all authors,

Dr Mtisunge Kachingwe

**Academic editor:**

**1. Please ensure that your manuscript meets PLOS ONE's style requirements, including those for file naming.**

The manuscript has been amended to meet the PLOS ONE style requirements

**2. Thank you for providing the date(s) when patient medical information was initially recorded. Please also include the date(s) on which your research team accessed the databases/records to obtain the retrospective data used in your study.**

Thank you the following information has been added to the materials and methods section.

In December 2017, the department created an electronic database of all admissions, including data entered from the paper records for patients from 1^st^ January 2017 and prospectively from December 2017. The research team accessed the database July 2019

**3. We note that the grant information you provided in the ‘Funding Information’ and ‘Financial Disclosure’ sections do not match. When you resubmit, please ensure that you provide the correct grant numbers for the awards you received for your study in the ‘Funding Information’ section.**

Thank you. In the funding information we have provided the names of the organizations from which we received funds. At receipt of funding we were not furnished with grant numbers from the organization. We hope this is acceptable.

We struggled to add more than one recipient in the funding information section of editorial manger. We would like to request that that the funding information and financial discloser read as follows: RKK and TB received grants for the study from Life Support Foundation (https://www.lifesupportfoundation.org/) and The Association of Anaesthetists (https://anaesthetists.org/) The funders had no role in study design, data collection and analysis, decision to publish, or preparation of the manuscript. We thank you for your assistance.

**4. In your Data Availability statement, you have not specified where the minimal data set underlying the results described in your manuscript can be found. PLOS defines a study's minimal data set as the underlying data used to reach the conclusions drawn in the manuscript and any additional data required to replicate the reported study findings in their entirety. All PLOS journals require that the minimal data set be made fully available. Upon re-submitting your revised manuscript, please upload your study’s minimal underlying data set as either Supporting Information files or to a stable, public repository and include the relevant URLs, DOIs, or accession numbers within your revised cover letter. For a list of acceptable repositories, please see** [**http://journals.plos.org/plosone/s/data-availability#loc-recommended-repositories**](http://journals.plos.org/plosone/s/data-availability#loc-recommended-repositories)**. Any potentially identifying patient information must be fully anonymized. Important: If there are ethical or legal restrictions to sharing your data publicly, please explain these restrictions in detail. Please see our guidelines for more information on what we consider unacceptable restrictions to publicly sharing data:** [**http://journals.plos.org/plosone/s/data-availability#loc-unacceptable-data-access-restrictions**](http://journals.plos.org/plosone/s/data-availability#loc-unacceptable-data-access-restrictions)**. Note that it is not acceptable for the authors to be the sole named individuals responsible for ensuring data access. We will update your Data Availability statement to reflect the information you provide in your cover letter.**

Thank you. The Authors share the belief that sharing data fosters scientific progress. In this case the data primarily belongs to the department anaesthesia at Queen Elizabeth central hospital. We the researchers had to seek permission from the department and ethical committee to access the database and at that time permission did not extend to sharing it publicly. Data is accessible upon request. The department would like to review each request on a case by case basis.

**5. Please amend the manuscript submission data (via Edit Submission) to include author Singatiya Stella Chikumbanje.**

Thank you. We have amended this.

**6. Your ethics statement should only appear in the Methods section of your manuscript. If your ethics statement is written in any section besides the Methods, please delete it from any other section.**

Thank you. The ethics section now only appears in the methods sections.

**7. Please upload a copy of Figure 2, to which you refer in your text on page 22. If the figure is no longer to be included as part of the submission please remove all reference to it within the text.**

Thank you. Figure 2 is no longer included as part of the submission. Reference to it has been removed within the text.

**Reviewer #1**:

**Thank you for asking me to review the manuscript, “Factors associated with poor outcomes in patients in an Intensive Care Unit in a tertiary hospital in Malawi.” The study covers an understudied and important topic of mortality prediction in critically ill patients from sub-Saharan Africa. The manuscript is well written and a nice contribution to the field. However, the authors may wish to consider the following.**
We thank the reviewer and have modified the manuscript to address the points made. We believe that the manuscript is now more readable, more informative, and its conclusions more useful to the public
**1. What is meant by “poor outcomes?” Since the study was designed to identify predictors of death in the ICU, would it not be better to explicitly state this in the title?**

Thank you. The title has been amended to – Factors associated with in-hospital mortality of patients admitted to an Intensive Care Unit in a tertiary hospital in Malawi

**Methodology
2. What was the reason for the study design that included both retrospective and prospective data collection? This study design likely had an impact on the amount of missing data, which likely influenced the results of the analysis.**

Thank you for your comment. We have included all the patients from the departmental database, which includes those entered retrospectively from January to November 2017. We believe the study is stronger for the inclusion of these additional patients. We have added text in the material and methods to clarify this.
**3. The authors state that data were collected from within one hour of admission to the ICU. Can they provide data regarding time from admission as this is time frame used to derive most of the severity scores tested?**

The vital signs documented were the first vital signs recorded on admission to the ICU, and the severity scores were based on these vital signs, not on admission to hospital. We do not have data on time of admission to hospital. Please let us know if this answers your query, or if we have misunderstood.
**4. Please provide more details about what imputation strategy was used and why the strategy was chosen.**

We have provided more details in the data analysis section in the material and methods.

**5. A sensitivity analysis is described based on analysis of patients with complete data; however, I cannot find the results of the sensitivity analysis in the Results section of the manuscript. Was the sensitivity analysis done?**

Apologies. We have now included the sensitivity analyses in supplementary table 1
**6. The greatest limitation of the study is found in the secondary analysis of severity scores. In this analysis, only sens/spec/ppv/npv was assessed using binary cutoffs from the scores. However, this analysis misses an opportunity to better assess the scores for their ability to discriminate for the outcome of death via the calculation of area under the receive operating characteristic curve. AUCs provide a better assessment of the overall discrimination ability of the tests. Using binary cutoffs reduces statistical power and provides a limited assessment particularly when the cutoffs used are essentially arbitrary and not validated in the population being analyzed, as in this case. Furthermore, the argument that clinicians in LMICs are not capable of calculating simple scores does not carry much water, particularly in ICUs and when smart phones and smart phone apps, which could calculate scores, are so ubiquitous in LMICs including those in Africa. Cutoffs can be useful, but individual clinicians need to assess and validate their priorities for maximizing sens/spec before assessing specific binary cut-offs for clinical use. As well, ppv/npv are dependent upon prevalence of the outcome of the population. Accordingly, if binary cut-offs are to be included, they should not be limited to arbitrary cutoffs, but instead a table should be provided with columns for sens/spec/ppv/npv and rows for each severity risk score starting from 0 to the maximum calculated score. As well, AUCs should be calculated for each severity risk score and for the assessment of the aggregate of single vital sign abnormalities.**
We agree that this is a really important issue and AUROCs can provide additional information. Indeed, in previous work we have studied AUROCs (for example Kayambankadzanja RK et al. The Prevalence and Outcomes of Sepsis in Adult Patients in Two Hospitals in Malawi. The American journal of tropical medicine and hygiene 2020, Baker T, et al. Single Deranged Physiologic Parameters Are Associated with Mortality in a Low-Income Country. Critical care medicine 2015; 43(10): 2171-9.)

However, for this study, we made the a-priori decision to use binary cut-offs for the scoring systems. This is due to our clinical and systems-based experience that in low-resourced environments with few staff, calculating and using compound scores is not feasible. In the settings where we have worked, including in the hospital in this study, when we have attempted to introduce compound scoring systems we have seen calculation errors, misclassifications, and after some time the scores are not used at all. Additionally, compound scores are unable to indicate potential appropriate treatments, in contrast to single severely deranged vital signs such as suggesting optimizing oxygen therapy when hypoxia is identified. Our aims in this study were to identify factors associated with in-hospital death and to study a binary score (single severely deranged vital signs) as we hypothesized that it could be useful and feasible to use, and compare it to other scores that have been converted to binary scores. The comparison of scoring systems was not the main aim of the study, and we are reluctant to add additional tables and performance measures as it could distract away from the main aim of the study.

We have added text about this in the discussion. We hope this is reasonable

**7. An additional significant limitation of this study is the application of severity risk scores to a heterogeneous clinical population that is different from the original derivation cohorts of the severity risk scores. In this study, the population was almost 50% post-surgical and clinically heterogeneous, which does not reflect the derivation population cohorts of the severity risk scores. Also, the scores were calculated at the time of admission to the ICU, not necessarily at the time of admission to hospital. Can the authors compare the performance of scores calculated at admission to hospital to those calculated at the time of admission to the ICU? Can the authors stratify the analyses based on medical vs surgical patients and whether data were retrospectively or prospectively collected? Also many patients were transferred from other hospitals so their physiology may already have been altered by preceding resuscitation which would impact the performance of severity risk scores. All of these issues should be detailed as limitations of the study in the Discussion**

Thank you for these good comments. We have added information to the discussion about these points.

We do not have data on the patients’ conditions on admission to hospital.

We have added tables 4 and 5 which show results of the models after stratification based on medical vs surgical patients

We have also added supplementary tables 5 & 6 showing the results after stratification based on prospective and retrospective data

The methods and results sections have been updated to report on these further analyses

**Results**
**8. Please provide a table of % missingness for each clinical variable/predictor used in the analyses. Please also provide data regarding how many patients were excluded from the analysis due to missing outcome data.**

Table with percentage of missing data has been added as a supplementary table 4

Two patients were excluded from analysis due to missing outcome data (start of results section)
**9. Please provide data for the pre-planned sensitivity analysis of patients with complete data**
Thank you. This is in supplementary table 1

**10. As above, please provide results of AUCs for severity risk scores and aggregate abnormal vital signs**

Thank you for your comment. We have responded in the above comment about AUROCs. We hope this is reasonable and acceptable.
**11. As above, please provide results for sens/spec/ppv/npv for each calculated score of the different severity scores starting at ‘0.’**

Thank you. We have responded to this in the above comment.
**12. For the assessment of individual risk factors for death, why was age categorized as < or >50 years? This seems to be an arbitrary cutoff which will lead to decreased statistical power. Age should probably be analyzed as a continuous variable.**

Thank you for the comment. A decision to convert age to binary was made a-priori for feasibility reasons and in keeping with the rest of the factors, even though we agree some statistical power is lost. Malawi has a young population hence the a-priori decision to categorize with a cut of 50.
**13. Consider creating a figure with a line graph of %mortality plotted against severity risk score results and a similar figure with OR 95%CI plotted against risk score results.**

Thank you for this recommendation. For this study we did not analyze for severity risk scores. We hope we understood the comment and that is acceptable.

**Reviewer #2:** 
**My primary comments are related to the data collection and definition of variables and the secondary aim (comparing predictive value of severity scoring systems within this cohort).**

We thank the reviewer and have modified the manuscript to address the points made. We believe that the manuscript is now more readable, more informative, and its conclusions more useful to the public
**Methodology**
**1. Additional details on data extraction would be helpful. What prospective data were extracted using the data sheet? What variables were abstracted from retrospective chart review? How was time of ICU admission determined? If multiple sets of vital signs were recorded within the first hour of ICU admission, which set of vital signs was included in analyses? How were laboratory values extracted? Were laboratory studies only included if they were drawn within the first hour of admission?**

Thank you for your comments. We have added additional details to the materials and method section, data collection section. All patients admitted to the ICU during the study period were included as study participants. In December 2017, the department created an electronic database of all admissions, including data entered from the paper records for patients from 1^st^ January 2017 and prospectively from December 2017. The prospective data were collected by the nurses immediately at admission or within 1 hour of admission if there were logistical delays in documentation, using a paper-based data collection tool. The data collection tool collected data on demographics, diagnosis, and ward from which they were admitted, vital signs, lab investigations and interventions received. The data extracted from the patients admitted between January and November included the first vital signs recorded during the first hour of admission to the ICU. Lab investigation results were obtained from the file. Data collection was supervised for quality in the ICU and double-data entered into the database. Follow-up of patients continued on the wards until hospital discharge or death. The primary endpoint for the study was in-hospital death. Patients lacking data on hospital outcome were excluded from analysis.

**2. The authors state that data were extracted from the departmental electronic database of all ICU patients” and “the data were collected prospectively from December 2017 by the nurses at admission or within 1 hour of admission, using a paper-based data collection tool.”**
**a. In the Data Management section, it is not clear if the variables of interest are restricted to the first hour of admission. For example, for a patient to be considered to have received mechanical ventilation in data analyses, did they have to be ventilated during the first hour of admission or did they count ventilation at any point during ICU admission?
b. Was retrospective data collection restricted to the first hour of admission?**

Thank you. This has been clarified in the data management section. The variable data was obtained from the data collection tool in which information was recorded within an hour of admission.

We have now clarified this in the data collection section. The retrospective data was also restricted to admission or within 1 hour of admission
**3. Lines 128 to 137: The section on comparison scoring systems (e.g. UVA, NEWS, TropICS) would benefit from additional clarification and explanation.**

A description of the models is now provided in supplementary table 2.

**a. The primary outcome of this study was in-hospital mortality. However, many of the comparison scoring systems were derived and validated to predict different outcomes (e.g. 24 hour mortality, ICU mortality, 3 day mortality) among different populations (e.g. patients on medical wards) from the study population. This should be acknowledged and justified.**

Thank you for this comment. We agree with the reviewers on this observation and have acknowledged this in the discussion. Assessments of the performances of the severity scoring systems has limitations as the population in this study had different case-mix characteristics and was exclusively an ICU population

**b. The authors state that “models were chosen for their greater potential feasibility in low resourced settings.” If this is the case, why did the authors select the NEWS instead of the MEWS which has been studied in Uganda? (PLoS ONE 11(3):e0151408. doi:10.1371/journal.pone.0151408)**

Thank you for your comment. We opted for NEWS as it more widely used. There are some studies that suggest that NEWS is better for example; Smith et al The ability of the National Early Warning Score (NEWS) to discriminate patients at risk of early cardiac arrest, unanticipated intensive care unit admission, and death, Resuscitation 84 (2013) 465–470

**c. The authors cite the Modified-MPM from Rwanda later in the manuscript, but do not include it as a reference scoring system. What was the reason for this?**

Thank you. We considered use of MPM score from Rwanda however we opted not to use it as we anticipated challenges to collect data on the variable of “confirmed or suspected infection”.

**Results**
**4. No information is provided about the number of records with missing data which were imputed as normal. Also no information provided about the results of the sensitivity analysis discussed in line 125.**

Table with amount of missing variable has been added as supplementary table 4

5. **The relatively low odds ratio for CPR is very interesting. Additionally, the proportion of patients receiving CPR within the first hour of ICU admission is quite high (20%). Additional information about how receipt of CPR was defined for the purposes of these analyses as well as additional data about the patients who received CPR would help interpret this finding.**

Thank you. We have amended the materials and method (data analysis) section to define CPR. Cardiopulmonary resuscitation consisted of advanced life support (ALS) to patients who suffered a cardiac arrest. (10) Cardiac arrest was defined in the Utstein style as “the cessation of cardiac mechanical activity confirmed by the absence of a detectable pulse, unresponsiveness, and apnea (or agonal respirations)”

The discussion section has been amended as follows

A high proportion of our patients (20%) received cardiopulmonary resuscitation. Majority were surgical patients admitted from theatre. This may reflect the illness severity of the patients on admission to the ICU as well as the quality anesthetic and surgical services. Thirty-nine percent of the patients in this study that underwent cardiopulmonary resuscitation survived hospital admission. A possible reason for the high success rate may be the young population of patients (median age 31) and few comorbidities such as diabetes mellitus, hypertension or cardiac disease. In the United States, the mean age of patients with in-hospital cardiac arrest is 66 years (39). Alternatively, a large proportion of the cardiac arrests may have been due to reversible causes, such as a lack of timely identification of an acute deterioration, equipment failure, challenges transporting ill patients from admitting ward to the ICU or other potentially avoidable reasons
**Discussion**
**6. Line 261: I do not think the analyses support broad conclusions about “the predictive performance” of the models. Many of the models were evaluated for their ability to predict outcomes they were not designed to predict. This conclusion should be more qualified**.

Thank you. The text and the conclusion have been modified.

**7. The limitation that some data were collected in a retrospective manner should be addressed.**

Thank you. This has been added to the discussion section.

**Methodology**

**8. Line 72: The authors refer to the “main” ICU as the site of data collection. Is information available on the other ICUs in the facility and the types of patients they admit? This would help to understand any potential biases associated with limiting inclusion criteria to one of multiple ICUs at the facility.**

The hospital has two ICUs. The second one recently established caters for pediatric patients and was not included in this study. Occasionally the adult ICU will admit pediatric patients when the pediatric ICU is full. We have changed from “main” to “adult” in the manuscript and clarified in the manuscript.

**9. Line 133: The authors include a citation for a commentary on the NEWS but not for the primary study and the TOTAL score manuscript should be cited here but is not cited until later in the manuscript.**

Thank you for this observation. The citing of TOTAL has been amended.

For NEWS the following article is cited as Royal College of Physicians. National Early Warning Score ( NEWS ) - Standardising the assessment of acute-illness severity in the NHS. Report of a working party. 2012. 47

We hope we understood this comment correctly.
**10. Line 143: Authors state that “each patient’s qSOFA, UVA, NEWS, MIME and TROPICS scores were calculated from their vital signs.” However, UVA and TropICS include non-vital sign data.**

Amended the terminology to variables

**Results
11. Line 157: “Other hospitals” is listed as a source of admissions to the ICU but this category is not included in Table 1.**

Table has been amended to say other hospitals

**12. Given the patient population is very heterogenous the authors may want to consider reporting odds ratios by the subgroups of surgical and non-surgical patients.**

Thank you. We have added tables 4 and 5 which report results based on surgical and medical patients.
