# Supplementary material for: Factors associated with in-hospital mortality of patients admitted to an intensive care unit in a tertiary hospital in Malawi
Source: PLoS One. 2022 Sep 30;17(9):e0273647. doi: 10.1371/journal.pone.0273647 (PMC9524689; doi:10.1371/journal.pone.0273647)
Supplement: Attachment — Submitted filename: Response to Reviewers 2.0.docx [file pone.0273647.s010.docx]

Dr Mtisunge Kachingwe
Queen Elizabeth Central hospital
P.O Box 95, Blantyre, Malawi

13/07/2022

Dear Editorial board members,

**RE: Response to reviewers**

We thank the two reviewers for their comments on our manuscript titled “Factors associated with in-hospital mortality of patients admitted to an Intensive Care Unit in a tertiary hospital in Malawi”. Below is our response to each point raised by the reviewers. We hope that we satisfyingly addressed them and that the manuscript will be now suited for publication.

Sincerely,

On behalf of all authors,

Dr Mtisunge Kachingwe

**Reviewer #1:**

**The authors have made a careful evaluation of the reviewer comments and have responded with appropriate revisions to the manuscript. However, concern remains about the use of cut-offs for the severity risk scores. Given the use of scores in a heterogeneous and unvalidated ICU patient population, the authors should provide more information about the performance of the scores. Sens/spec/ppv/npv can go up or down depending on the cut-off used and depending on the priority of the clinician. For example, in the data provided, NEWS >/=7 has a high sens and low spec; whereas, qSOFA >/=2 has low sens and high spec. Presumably, these could be reversed by simply adjusting the cut-offs for each score. Since the cut-offs are arbitrary and we don't know the optimal cut-off for each score evaluated in this population, the authors should provide sens/spec/ppv/npv for each value within each severity risk score so they can be appropriately evaluated.**

Thank you for this comment! We have further analysed the scoring systems using every possible cut-off for a critical score and present all the performance measures in Supplementary Table 7.

**To use the prior example again, perhaps 7 and 2 are simply not the correct cut-offs (if one exists) in this population for NEWS and qSOFA, respectively. The overall AUC for each score would also be useful information and should be reported. If the authors are unable or unwilling to provide these data, then they should consider not including the secondary aim of evaluating severity risk scores.**

Thank you for this. The AUCs for each scoring system has now been added to the results section.

**Finally, the large amount of missing GCS data should be further emphasized as a limitation particularly for GCS evaluation alone and for scores which include GCS, i.e. UVA. Accordingly, each risk score should be added to Supplementary Table 1 to show the effect of missing data on the performance of the risk scores. Ideally, this would include how AUC changes.**

We thank you for your comment. We have added text to the discussion to highlight the missing data, especially for GCS and capillary refill time. We have added a Supplementary Table 8 to show the performance of the severity scores with just complete data.

**Reviewer #2: Dr. Kachingwe and colleagues have carefully revised their manuscript.

Major comments**
 **Discussion
1) The second part of the Discussion is dedicated to deranged vital signs and severity scores as predictors of in-hospital mortality. While I agree with the authors’ central argument—that the presence of one or more severely deranged vital signs on admission to an ICU in Malawi is a feasible prognostic marker for increased risk of in-hospital death—I think this conclusion needs further qualification due to the heterogeneity of the study population and the rate of missing data. Lines 331 to 339 provide an excellent summary as well as clear and helpful context. I think this is the core of the part of the discussion and would consider streamlining the rest of this section.**

Thank you for these comments. We have qualified our conclusions in the discussion. And we agree that the discussion is long, however it is hard to streamline further than has already been done, as several parts were requested to be added during the review process. I hope that is ok.

**Study population heterogeneity: The authors state that “****assessments of the performances of the severity scoring systems has limitations as the population in this study had different case-mix characteristics.” However, this also seems to be applicable to single deranged vital signs. I would consider acknowledging this and including discussion on how this limitation may have influenced the results.**

Thanks, we have amended this to clarify that it concerns all results.

**b. Missing data: Please expand on the limitation that “there are some missing data”. There were significant rates of missing data, for the variables capillary refill time and Glasgow coma scale in particular. By providing the two sensitivity analyses in supplementary table 1 (i.e., case wise deletion of missing data and imputed abnormal data), the authors do an excellent job helping the reader to understand the potential impact of missing data on their estimates. I think the manuscript would benefit from a more detailed discussion in the text comparing estimates from the primary analysis with the two sensitivity analyses in table S1.**

Thank you, this has been added.

**1) The NEWS score and any severely deranged vital sign had very similar point estimates in the primary analysis (Table 3). It therefore strikes me as inconsistent to say that “performances of the models were too low to be clinically useful by themselves for individual patient decisions” and to also suggest that severely deranged vital signs have clinical utility.**

Thank you for this comment. We have clarified that we meant that all the scoring systems, including single severely deranged vital signs, do not have good enough performances to be used as sole markers for individual patient decisions, but that single severely deranged vital signs could be useful as a marker of an increased risk of death together with other clinical information.

**Minor comments

1. The authors state: “critical illness as defined by the binary cut-offs in each of the severity models showed a significant association with in-hospital mortality.” However, not all of the severity model binary cut-offs had a significant association with in-hospital mortality.**

Thank you. The sentence has been amended.

**2. Tables 4 and 5: Percentages are missing from the first data column**

Thank you, this has been added

**3. Supplementary table 1: What is the total number of patients with complete data included in these analyses?**

Thank you for this observation. The total number of patients with complete data per each variable/factor has been added to the supplementary table 1.

**4. The manuscript needs additional copy editing**.

The manuscript has been proof-read again by English language experts and changes made.
